# Network Redundancy Analysis of Effective Brain Networks; a Comparison of Healthy Controls and Patients with Major Depression

**DOI:** 10.1371/journal.pone.0060956

**Published:** 2013-04-18

**Authors:** Lutz Leistritz, Thomas Weiss, Karl-Jürgen Bär, Fabrizio De VicoFallani, Fabio Babiloni, Herbert Witte, Thomas Lehmann

**Affiliations:** 1 Institute of Medical Statistics, Computer Sciences and Documentation, Jena University Hospital, Friedrich Schiller University Jena, Jena, Germany; 2 Department of Biological and Clinical Psychology, Friedrich Schiller University Jena, Jena, Germany; 3 Department of Psychiatry and Psychotherapy, Jena University Hospital, Friedrich Schiller University Jena, Jena, Germany; 4 Department of Physiology and Pharmacology, University "Sapienza", Rome, Italy; 5 Neuroeletrical Imaging and Brain Computer Interface Laboratory, IRCCS Fondazione Santa Lucia, Rome, Italy; 6 Centre de Recherche de l'Institut du Cerveau et de la Moelle Épinière (CRICM), Hôpital de La Pitié-Salpêtrière, Paris, France; University of California, San Francisco, United States of America

## Abstract

This study investigated electroencephalographic correlates in chronically depressed patients compared to healthy controls using intracutaneously applied electrical pain stimulus, to better understand the interaction between pain processing and depression. A close interaction between pain and depression is generally recognized although the precise mechanisms are not yet fully understood. The present study focuses on the hypothesis that effective brain connectivity in major depression patients is altered. Multifunctional interactions between brain regions represent a robust index of effective interactions within the brain, and can be quantified by network redundancy. Thus, structural network differences between 18 normal controls and 18 major depression patients before as well as during the processing of moderately painful intracutaneous electrical stimuli were investigated on the basis of network redundancy differences. In our sample, both patients and control subjects exhibit comparable network redundancies before stimulus application. Caused by the stimulus, there is a global increase of network redundancy in both groups. This increase is diminished in the group of major depression patients. We found clear differences between patients and controls during the stimulus processing, where the network redundancy in normal controls is larger in comparison to patients. The differences might be explained by the fact that major depression patients are more restricted to the affective component of the processing. The well-established biasing to affective processing might suppress the somatosensory processing resulting in a lower number of connections within the considered network. This might then lead to a reduction in network redundancy during stimulus processing.

## Introduction

A close interaction between pain and depression is generally recognized although the precise mechanisms are not yet fully understood. Epidemiological studies report a mean prevalence rate of major depressive (MD) disorder in patients with chronic pain assessed in pain clinics of 52%, and a mean prevalence of pain in depressed patients of 65% [1]. Depressed patients generally describe more general physical symptoms such as abdominal discomfort or musculoskeletal pain [2] vs. localized pain sites.

Multiple brain regions such as the insular cortex, prefrontal cortex (PFC), anterior cingulate cortex (ACC), amygdala and hippocampus have been implicated in both MD and pain. Shared neurocircuitries and neurotransmitters may play a role in connecting the two pathophysiologies of depression and pain; in this complex interplay any alteration in brain activity or function caused by one disorder can affect the other. In addition, human imaging studies have shown that MD is associated with abnormally increased activation within an emotion-processing network that includes the extended amygdala and prefrontal cortex during the anticipation of negative images [3]. Related studies that have examined experimental pain processes in depressed patients have provided evidence that MD is associated with functional alterations of emotion-processing circuitry during the perception of pain [4]. Although a correlation between depression and pain has been accepted in the last few years, the underlying physiological background still remains unsolved. One hypothesis that the processing of noxious stimuli within the so- called “neuromatrix of pain” might be different was recently affirmed [5]. The authors found that the effective brain connectivity differs during the processing of nociceptive stimuli between MD patients and healthy controls (HCs). Moreover, stimulus-induced alterations of the connectivity pattern were considerably more pronounced in the HCs compared to MD patients.

The aim of the present study is to present a more complete description of structural network differences between HCs and MD patients. Such network (graph theoretical) approaches have achieved an increasing impact in computational neurosciences because topological network properties are closely related to optimal information processing and signal transmission between neural units [6]. In particular, the small-world phenomenon [7] is frequently used to describe topological network properties [8]–[11]. The small-world concept is closely related to shortest path lengths between network nodes. However, beyond shortest paths, existing longer pathways may be used to characterize complex connectivity patterns. The existence of multiple parallel pathways seems to be closely related to a concept of redundancy and robustness, which is thought to be a natural mechanism of the brain for enhancing the resilience to neural damage and dysfunction [12].

Based on the differing connectivity patterns found in the HCs and MD patients, particularly with regard to induced changes evoked by nociceptive stimuli, alterations of the network redundancy may be supposed because of observed modified reactions to painful stimuli in MD patients, and because that reactivity might be related to effective connectivity. In addition, network redundancy can be interpreted as multifunctional interactions between network nodes, and it represents a robust index of effective interactions between different brain regions. Therefore, the goal of this study was to reveal network redundancy differences between HCs and MD patients before as well as during the processing of moderately painful intracutaneous electrical stimuli. We expect interesting new insights into the relationship between chronic pain and depression. The present study directly follows up on the EEG experiments and the connectivity estimates published previously [5] and is based on the same experimental data.

## Materials and Methods

### Subjects

Eighteen patients (10 women, 8 men) with major depression (mean age ± standard deviation: 38.9±15.5 years) and 18 sex-and age-matched healthy control subjects (39.3±14.8 years) participated in the study. Patients were treated in a specialized psychiatric ward for mood disorders. Major depression was established by a staff psychiatrist according to DSM IV criteria using a structured interview [SCID, [13]]. Beck depression inventory (BDI) was also administered. BDI scores of patients ranged from 19 to 48 (29.4±9.7); scores of control subjects were all below five (2.1±1.5). All subjects were right-handed. Nine patients received antidepressive medication (5 patients received selective serotonin reuptake inhibitors SSRI; 4 patients norepinephrine and serotonin reuptake inhibitors NaSRI) while the remaining patients and all healthy subjects were free of any medication. Prior to the experiment detailed information on the aim and the procedures of the experiment was provided to each subject and written informed consent was obtained. The procedure was approved by the Ethics Committee of the Friedrich-Schiller-University (reference number 2282–04/08).

### Paradigm

Prior to the actual experiment, individual stimulus-response properties to intracutaneous electrical stimuli [14] were determined. Stimuli consisted of a bipolar rectangular pulse of 10 ms duration generated by a constant current stimulator (DS7H; Digitimer, Welwyn Garden City, UK). Stimuli were applied intracutaneously to the tip of the middle fingers of both the right and the left hand through isolated golden pin electrodes with a diameter of.95 mm and a length of 1 mm (for details of stimulation see [15], [16]). The pin was inserted into a small epidermal cavity of 1 mm diameter to about 1 mm depth and fixed with adhesive tape. The purpose of this preparation was to reduce skin resistance and thus the current necessary to elicit a pain sensation. A flexible stainless-steel electrode, fixed loosely around the first finger joint, served as a reference electrode. Subjects were grounded by using a broad, flexible, humid band electrode fixed around the wrist of the stimulated hand. Using a modified method of limits, 3 series of single electrical pulses with up- and down-going intensities were applied. Current intensity varied between 10 µA and 1 mA. Participants were requested to rate each electrical stimulus on a scale ranging from 0 to 6 (0 = no sensation; 1 = just perceived, not painful; 2 = clearly perceived, but not painful; 3 = low pain; 4 = moderate pain; 5 = strong pain, but tolerable; 6 = unbearable pain, see [17]). Pain threshold was defined as the intensity yielding a sensation described as a sharp painful pinprick, corresponding to a rating of “3”.

The mean intensity of the last two series of up- and down-going series to elicit an intensity rating of “4” was used in the experiment. One patient and two controls were excluded from this study because they did not answer to this series in the predicted way (increasing ratings with up-going intensities and decreasing ratings with down-going intensities) such that we were not able to determine a meaningful intensity rating of “4”. During the main experiment, ninety moderate painful electrical stimuli with a constant inter-stimulus interval of 3 s were administered intracutaneously to the left and right middle fingers. We used a constant inter-stimulus interval because it is known that subjects feel more confident in a situation when stimulus intensity and time point of stimulation are known compared to when these parameters are unknown. Thus, from the perspective of patients with major depression it seems more appropriate to use such a design. Finally, intensity ratings were obtained every 30 stimuli with short breaks in between electrical stimulations.

The EEG was recorded continuously using Neuroscan (now Charlotte, USA) amplifiers during the electrical stimulation from 60 electrodes, referenced to Cz, using a standard EEG cap (Easy Cap, Falk Minow Services, Germany) based on an extended International 10–20 system. Eye movements were also monitored. All electrode impedances were kept <5 kΩ. After analog filtering (0.1–100 Hz), the EEG was sampled at 500 Hz. The recordings were subsequently re-referenced to a linked ears reference. In this study, only data from 9 electrodes were processed: F3, Fz, F4, C3, Cz, C4, P3, Pz, and P4 according to the extended International 10–20 System of Electrode Placement. These electrodes were chosen because they are situated above important regions of pain processing, attention, and depression (frontal, central, and parietal brain regions). These areas alone do not cover the entire brain and some interesting areas of pain processing and depression, such as the insula or prefrontal cortices, were omitted. However, the intention of this study was to show the applicability of the methodology to the present type of data. Furthermore, we avoided utilizing adjacent electrodes because they are usually highly correlated, which is disadvantageous for any Granger Causality or PDC analysis on the basis of an autoregressive modeling of the underlying processes.

### Data Preprocessing

We used signal sections of 700 ms for the analysis: 700 ms pre stimulus for the pre-stimulus condition (700 ms before stimulus to stimulus onset) as well as 700 ms post stimulus for the post-stimulus condition (interval from stimulus onset to 700 ms post stimulus). In order to remove artifact contaminated single trials we used the potentials at the electrodes Fp1, AF7, AF1, AF5, Fp2, AF8, AF2, AF6, O1, O2, Oz, T7, T8, F7 and F8, because artifacts were most pronounced at these electrodes. These are most likely caused by eye movements and mastication muscle activity. A single trial somatosensory evoked potential (SEP) was excluded if the maximum absolute voltage at any of the above electrodes exceeded 200 µV in the considered time interval. This procedure resulted in the exclusion of three data sets since there were not enough, i.e. less than 25, artifact free trials left for a reliable analysis. Finally, 70.5 trials remained on average (standard deviation 13.1, minimum 27, maximum 89).

### Connectivity Analysis

In order to assess the effective connectivity between the nine nodes the generalized partial directed coherence [18] was applied. It is based on a multivariate autoregressive (AR) modeling of the measured signals. To estimate the model parameters we used a multi-trial multivariate least square estimator [19].

For each subject and condition the estimator was provided with a large set of associated trials, i.e. each single trial is considered as independent realizations of the same process. Finally, the multi-trial estimator yields one common AR parameter set for an entire set of single trials, instead of one estimation for each single trial.

An initial model order was determined according to Akaike’s information criterion (AIC) [20] and tuned to achieve coincidence between the parametric (AR related) and the Fourier power spectra, where both spectra were compared for various model orders. Finally, the smallest order, where all substantial frequency components were represented by the parametric estimation, was chosen. That is, it had to be ensured that the parametric spectrum reconstructs all distinctive spectral peaks in the frequency range of interest, where we considered only peaks with amplitudes exceeding the noise level by factor two in at least three adjacent frequency bins.

The tuning procedure considers that AIC criterion often results in an overestimated model order. A proper fine-tuning is necessary because the model order must be large enough to avoid severe biases while the efficiency of the estimates suffers from too large orders. Between both extremes, there is usually a broader range where different model orders result in identically identified directed interactions. Aiming at a group comparison of connectivity measures, constant model settings have to be used for all sample elements. Thus by means of the maximum of single order estimations, we selected a common model order of 40 for all data sets.

**Figure 1:**
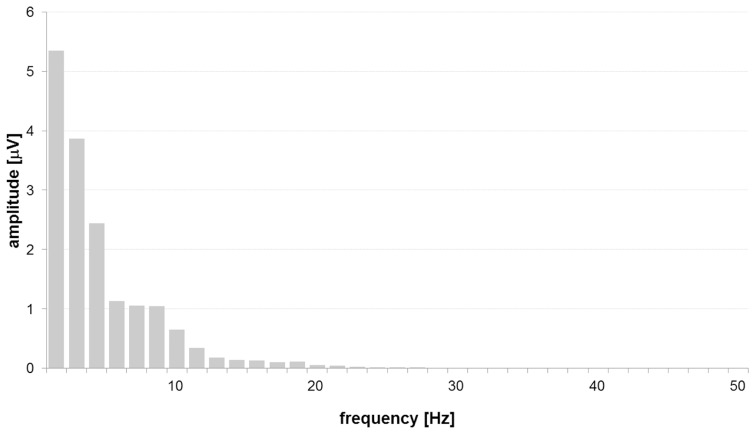
Amplitude spectrum of grand average data at Cz post stimulus.

We restricted our analysis to a frequency band with a high band power in order to work with a maximized signal to noise ratio. The frequency band was determined to be in the delta-, theta- and the alpha-bands (1 to 13 Hz) since the signal power is mainly situated in this frequency range (Figure 1). Other frequency bands haven’t been investigated due to low signal power. For a consolidated analysis we pooled the generalized partial coherences (gPDCs) of the corresponding frequencies to one quantity by averaging gPDCs of the frequency range of interest. Thus, for each of the 72 (i.e. 9^2^–9) possible directed interactions one quantity resulted. The range of pooled gPDC values was [0.01 0.89]. Four conditions were analyzed for each subject: pre stimulus/left middle finger, pre stimulus/right middle finger, post stimulus/left middle finger, and post stimulus/right middle finger, i.e. four data sets were available for every subject. Each data set was composed of multiple single trials, which were simultaneously processed to obtain one PDC matrix. Thus finally, exactly one interaction pattern was revealed for every subject and condition. A detailed description of the procedure may be found in [5].

**Figure 2:**
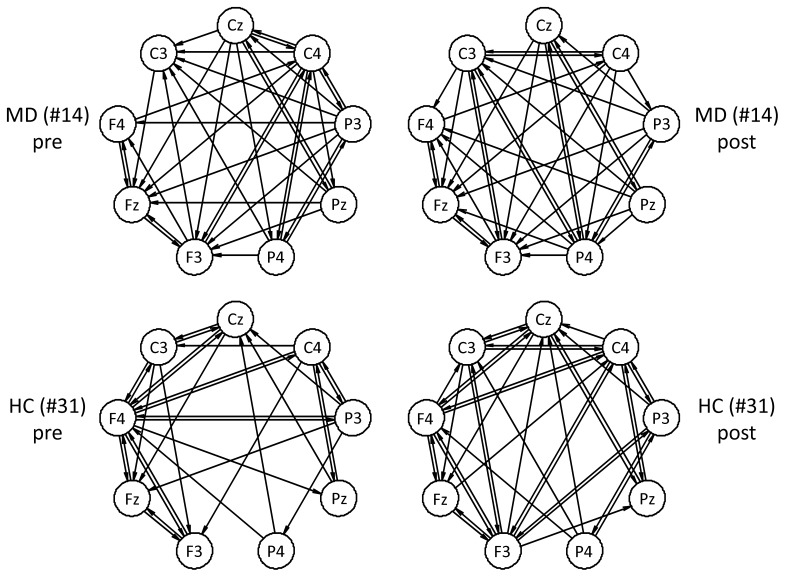
Some examples of identified directed networks (graphs) according to [**5**]. The upper row shows networks of an MD patient during the pre and post stimulus condition (left hand side stimulation). The lower row refers to a control subject accordingly. Here, for the sake of clarity the electrode positions are circularly arranged.

One of the most important questions is whether the pooled generalized partial directed coherence is significantly greater than the gPDC obtained when no interaction is present, which is defined as the null hypothesis H_0_. The distribution under H_0_ is analytically not known, thus it was constructed by the Bootstrap procedure introduced in [21]. Briefly summarized, this method represents a residual resampling, where the common starting points are the model residuals and the set of estimated autoregressive parameters 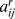, 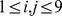, 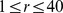. Assuming that the interaction from the *l* ^th^ to the *k* ^th^ process component should be tested, at first all parameters 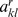 are set to zero. That modification effects an elimination of a possible interaction from process component *l* to *k*. Based on this modified AR parameter set and the resampled residuals from the original estimation (sampling with replacement), a resampled time series is generated, which is finally used to estimate one Bootstrap replication of the underlying test statistics under the null hypothesis “no interaction from process component *l* to *k*”. We used 1500 Bootstrap repetitions to estimate the test statistics distribution under H_0_. Because interactions in a network have to be considered as a whole, an α-adjustment is necessary. For all multiple test procedures, the Holm correction [22] with a multiple significance level of 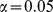 was applied to control the familywise error rate for all 72 hypotheses at α. Exemplary identified networks are shown in Figure 2. A drawback of this approach might be that a successful simulation of an AR process based on the estimated AR parameter set cannot be guaranteed. In this case, the significance threshold cannot be determined by this procedure. With respect to the entire sample, we detected this situation in 3.48% of all possible directed interactions. We registered and treated the connections as missing values in these cases.

### Imputation of Missing Values and Statistics

Despite the minor number of missing values, many networks were affected. This is caused by the fact that one network contains 72 edges, and a network as a whole is already affected, if at least one edge information is missing. Finally, only 12.5% of networks were not affected by missing values. Excluding patients with missing values from analysis (list wise deletion) reduces statistical power and, even worse, often leads to biased estimates [23].

In practice observed data often contain useful information for predicting the missing values. In such cases, the data are missing at random (MAR) and imputation procedures can exploit this information to obtain unbiased estimates and retain the full sample size. To account for the additional uncertainty due to imputation, missing data are replaced by *m* >1 simulated values (multiple imputation) yielding *m* completed datasets. Each dataset has been analyzed with standard statistical complete-data methods (i.e. mixed linear model) and results of each data analysis are combined according to Rubin’s rules for multiple imputation inference [24].

Fully Conditional Specification (FCS) is a multiple imputation method which does not require assumptions about the joint distribution of the variables (i.e. multivariate normality). Instead of the joint model, conditional distributions (regression imputation models) for each variable with missing values, involving all other variables as predictors, have to be specified. Imputation under FCS is done by iterating over all conditionally specified imputation models, each iteration consisting of one cycle through all variables with missing values (Gibbs sampling procedure) [25]. FCS can handle different types (binary, ordered, continuous) of variables and we used this flexible method to deal with missing values in our data set of directed graphs.

### Network Redundancy

The theoretical representation of a network is the graph. A graph consists of a set of *N* nodes and a set of links (connections) indicating the presence of some sort of interaction between the nodes. The adjacency matrix **A** contains the information about the connectivity structure of the graph and it has dimensions *N*×*N*. When a link connects two nodes *i* and *j*, the corresponding entry of the adjacency matrix *a_ij_* is equal to one, otherwise it equals zero. In a graph, a path is a sequence of nodes such that, from each of its nodes, there is a link to the next vertex in that sequence. Shortest paths represent one possible way in which two nodes in a graph can interact. Existing longer pathways can be generally taken into account when characterizing functional brain connectivity patterns [26]. The most intuitive way to compute all the possible paths in a graph is to count the total number of paths between the nodes, which is a NP-complete problem. Thus, a three-dimensional matrix **P** of size *N*×*N*×*L* may be determined, containing the number of all the possible paths of length *l* = 1, …, *L* in each node pair, where *L* is at maximum *N*–1. From this **P** matrix, the following characteristic measures can be defined:

The *scalar redundancy R_s_* is the total sum of the number of paths of any length *l* = 1, …, *N*–1, found between all the nodes, excluding the self-connections
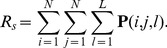
(1)


It represents the global level of network redundancy by means of a scalar number. The higher *R_s_* is, the higher the tendency is of the graph to exhibit multiple parallel pathways.

The *vectorial redundancy R_v_* is the total sum of the number of paths found between all the nodes, excluding the self-connections, with respect to each path length *l* = 1, …, *N*–1
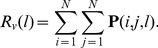
(2)


It represents the total level of network redundancy across different path lengths. The higher 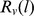 is, the higher the tendency of the graph is to exhibit multiple parallel pathways with a specific length *l*. In particular, 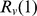 corresponds to the number of connections in the graph (network).

### Statistical Analysis

Because of multiple measurements of each subject (left hand side and right hand side stimulation, pre stimulus and post stimulus situation), a statistical method that considers the correlation structure has to be applied. An appropriate statistical approach is given by linear mixed models (LMMs) [27], where the dependent variables were specified by the scalar *R_s_* or vector redundancies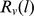, *l* = 1, …, 8, respectively. In detail, we used nine separate LMMs to estimate mean network redundancies
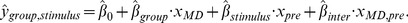
(3)


For different models, the symbol *y* is respectively substituted by *R_s_* or 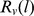. The group assignment (MD: 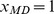, HC: 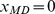) and the stimulus condition (pre: 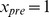, post: 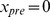) were used as fixed factors. In order to show different progressions between the HC and MD groups, we were particularly interested in the interaction of group assignment and stimulus condition. Thus, we included the corresponding interaction term in the LMM. Thereby, the corresponding indicator variable 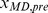 is exclusively equal to one for MD patients during the pre-stimulus condition. Furthermore, the side information (left, right) was used as repeated measurements. The inclusion of this variable is important to estimate the covariance matrix of subject-specific residual errors properly. In contrast to repeated measures ANOVA, which implies a compound symmetry covariance structure, LMMs provide more flexibility in modeling the residual covariance matrix. We assumed first-order ante dependence as a general covariance structure for the residuals in all LMMs, because this structure exhibited the best fit according to a restricted maximum likelihood estimation in comparison to other substantially suitable covariance structures, although a small number of parameters has to be estimated using this covariance structure.

All statistical analyses and the multiple imputation of missing values were performed by means of IBM SPSS Statistics 18. The common significance level was set to 5%.

## Results

**Figure 3:**
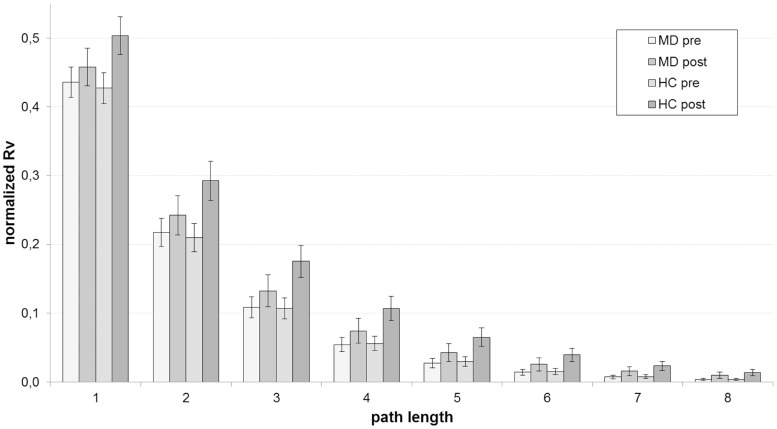
Mean normalized network redundancy estimates of the LMMs with their standard errors for different path lengths. Normalized network redundancy means that 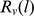
is normalized by the corresponding value of a complete (that is: fully connected) network consisting of nine nodes.

**Figure 4:**
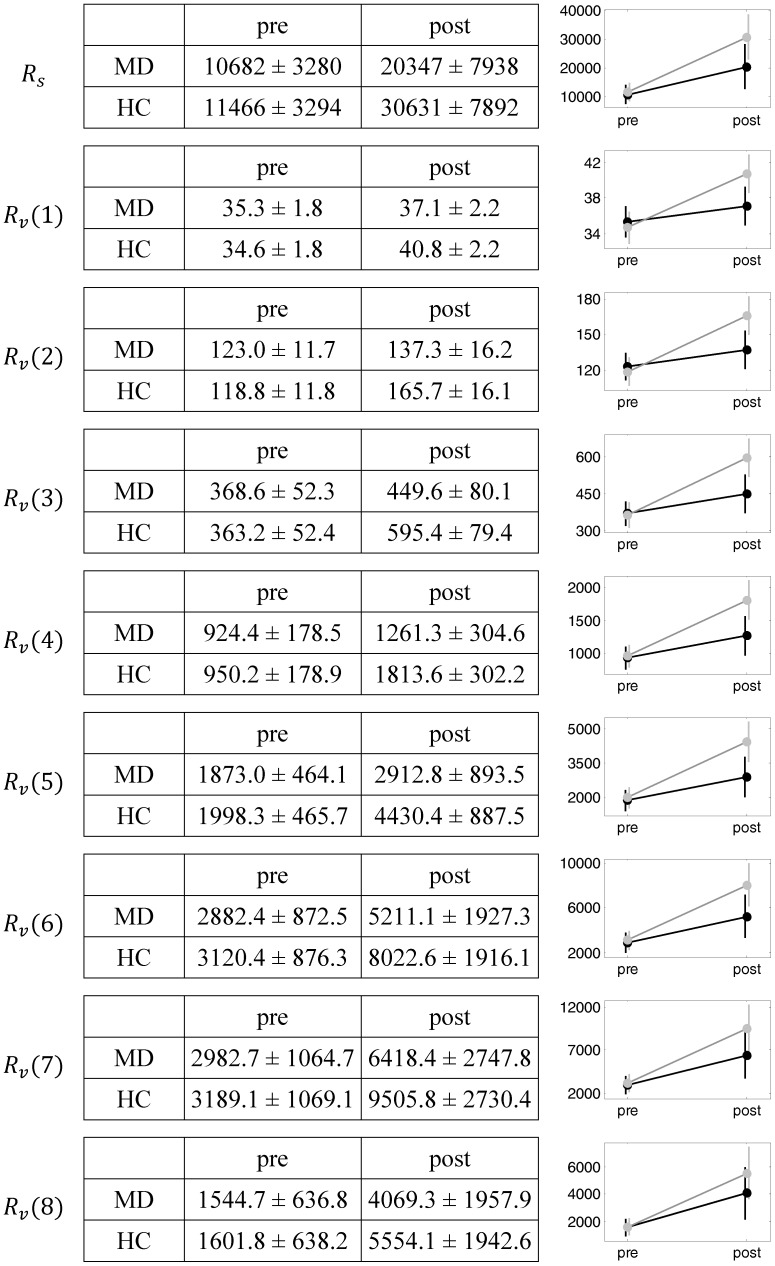
Mean network redundancy estimates of the LMMs with their standard errors. The right column depicts the mean values and their standard errors graphically. *R_s_* refers to the scalar network redundancy, 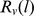, *l* = 1, …, 8, denote the vector redundancy measures for different path lengths.

Tables 1, 2, 3, 4, 5, 6, 7, 8 and 9 show the LMM’s regression coefficient estimates and their standard errors depending on different network redundancy characteristics. It is noticeable that a subject assigned to the MD group is provided with a reduction of the mean network redundancy independent of the considered redundancy measure (all regression coefficients of rows *β_group_* are negative). Furthermore, an analogous statement is applicable for the pre stimulus situation (all regression coefficients of rows *β_stimulus_* are negative). Neglecting the interaction, this implies that the stimulus resulted in an increase of all considered network redundancy measures *R_s_* and 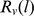, *l* = 1, …, 8. That effect is significantly independent of the network redundancy measure considered. In contrast, the regression coefficient *β_inter_* referring to the interaction of group assignment and stimulus condition is always positive; i.e., the combination of an MD subject with the pre stimulus situation yields an increase of the mean network redundancy independent of the considered redundancy measure. Bundling all three statements, the difference between the MD and HC group is diminished in the pre stimulus situation, while the main difference between the two groups appears in the post stimulus condition. This effect is especially prominent in the case of 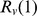 (number of directed interactions), 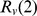, and 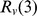, where it is even significant for 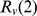. These findings are also found in Figure 3 and Figure 4. They show mean network redundancy estimates of the LMMs with their standard errors. In our sample, both MD and HC subjects begin the pre-stimulus interval at comparable levels. As shown by the statistics, there is a global increase of network redundancy caused by the stimulus. This increase is pronounced in the HC group. In particular, this relationship can be observed for vector redundancy measures with low path lengths (e.g. from one to three).

**Table 1:** LMM estimates of the regression parameters with respect to *R_s_* and their standard errors (bold: significances).

Parameter	Mean ± Standard Error	P-value
*β_0_*	30631±7892	
*β_group_*	−10284±1181	*p* = 0.358
*β_stimulus_*	−19165±6446	***p*** ** = 0.003**
*β_inter_*	9501±9182	*p* = 0.301

**Table 2:** LMM estimates of the regression parameters with respect to 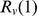 and their standard errors (bold: significances).

Parameter	Mean ± Standard Error	P-value
*β_0_*	40.76±2.19	
*β_group_*	−3.70±3.11	*p* = 0.234
*β_stimulus_*	−6.12±1.57	***p*** **<0.001**
*β_inter_*	4.37±2.23	*p* = 0.051

**Table 3:** LMM estimates of the regression parameters with respect to 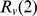 and their standard errors (bold: significances).

Parameter	Mean ± Standard Error	P-value
*β_0_*	165.7±16.1	
*β_group_*	−28.43±22.83	*p* = 0.213
*β_stimulus_*	−46.95±11.30	***p*** **<0.001**
*β_inter_*	32.70±16.10	***p*** ** = 0.042**

**Table 4:** LMM estimates of the regression parameters with respect to 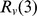 and their standard errors (bold: significances).

Parameter	Mean ± Standard Error	P-value
*β_0_*	595.4±79.4	
*β_group_*	−145.8±112.7	*p* = 0.196
*β_stimulus_*	−232.2±56.5	***p*** **<0.001**
*β_inter_*	151.2±80.7	*p* = 0.061

**Table 5:** LMM estimates of the regression parameters with respect to 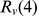 and their standard errors (bold: significances).

Parameter	Mean ± Standard Error	P-value
*β_0_*	1814±302	
*β_group_*	−552.3±428.8	*p* = 0.198
*β_stimulus_*	−863.3±221.2	***p*** **<0.001**
*β_inter_*	526.4±315.8	*p* = 0.095

**Table 6:** LMM estimates of the regression parameters with respect to 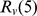 and their standard errors (bold: significances).

Parameter	Mean ± Standard Error	P-value
*β_0_*	4430±887	
*β_group_*	−1518±1285	*p* = 0.228
*β_stimulus_*	−2432±676	***p*** **<0.001**
*β_inter_*	1392±964	*p* = 0.149

**Table 7:** LMM estimates of the regression parameters with respect to 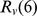 and their standard errors (bold: significances).

Parameter	Mean ± Standard Error	P-value
*β_0_*	8023±1916	
*β_group_*	−2811±2715	*p* = 0.300
*β_stimulus_*	−4902±1527	***p*** ** = 0.001**
*β_inter_*	2573±2176	*p* = 0.237

**Table 8:** LMM estimates of the regression parameters with respect to 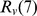 and their standard errors (bold: significances).

Parameter	Mean ± Standard Error	P-value
*β_0_*	9506±2730	*p*<0.001
*β_group_*	−3087±3867	*p* = 0.425
*β_stimulus_*	−6317±2278	***p*** ** = 0.006**
*β_inter_*	2881±3244	*p* = 0.374

**Table 9:** LMM estimates of the regression parameters with respect to 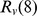 and their standard errors (bold: significances).

Parameter	Mean ± Standard Error	P-value
*β_0_*	5554±1943	*p* = 0.004
*β_group_*	−1485±2751	*p* = 0.589
*β_stimulus_*	−3952±1692	***p*** ** = 0.020**
*β_inter_*	1428±2406	*p* = 0.553

## Discussion

This study investigated electroencephalographic correlates in chronically depressed patients compared to healthy controls using intracutaneously applied electrical pain stimulus, to better understand the interaction between pain processing and depression. We have investigated network redundancy and found robust changes for the stimulus condition. Network redundancy is significantly lower during the pre-stimulus time window compared to the time window during which the specific nociceptive information is processed. More importantly, we found clear differences for these changes between MD patients and HCs as evidenced by a lower increase of redundancy for MD patients compared to HCs. These differences were pronounced for smaller network redundancies and reached significance for 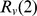. Unexpectedly, there was no significant difference between MD patients and controls during the pre-stimulus time window.

We found a clear increase in all redundancy parameters for the comparison of the network structures of the time window when comparing the pre and post stimulus condition. An explanation might be proposed with respect to the processing of noxious stimuli within the neuromatrix of pain [28]. It is well accepted that the processing of a noxious stimulus involves a number of structures (thalamus, primary and secondary somatosensory cortex), insula, anterior cingulate cortex, prefrontal cortex etc. [29]–[31] including several functional components, e.g. somatosensory or affective components [32]–[34].Thus, the processing of the noxious stimulus is associated with more intensive interactions of these components involving the aforementioned structures. Indeed, we found an increase of overall connectivity 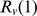 in the stimulus condition. In addition, we also reported an increased number of interaction trails in terms of redundant alternative pathways which reflect a global tendency of the brain to strengthen the short and long range interactions between the regions of the brain in response to the painful stimulus.

Importantly, we found clear differences between MD patients and HCs for the time period after the noxious stimulation. In particular, our data demonstrates for the first time that the redundancy in HCs is larger in comparison to patients during the processing of the noxious stimulus. An explanation might be proposed with respect to the processing of noxious stimuli within neuromatrix of pain; the processing in MD patients may be more restricted to the affective component of the processing as indicated by the increased activation in the anterior insula during heat pain perception in patients with MD in comparison to controls [4]. The well-established biasing to affective processing might suppress the somatosensory processing resulting in a lower number of connections within the considered network. This might then lead to a reduction in redundancy during the processing of the stimulus in the MD group.

Network redundancy was previously studied by De Vico Fallani et al. [26] who compared patients with spinal cord injury and healthy controls during the attempt to move one’s feet. This is the only other study to the best of our knowledge which has investigated network redundancy parameters in patients and control subjects. The results of this work demonstrated differences in redundancy between groups depending on the frequency band. Both studies are highly suggestive that multiple pathway analysis is useful for detecting changes in network structures by exploiting all the available information contained in paths of any length. Such investigative methods might shed light on the hypothesis of altered information processing in different populations.

We did not observe significant differences between MD patients and controls during the pre-stimulus time window, although we also expected altered redundancies in the pre-stimulus network structure due to the affective bias of MD patients. There are several explanations for this finding. Possibly, the extent of processing and redundancy due to the expectation of the stimulus is the determining activity in the network during this period of time, thus it is less influenced by the affective bias. An alternative interpretation might be that the expectation of a nociceptive stimulus evoking the sensation of pain is associated with a negative affective processing in HC subjects. In addition, it might be possible that the applied methods discriminate groups more sensitive after a defined and arousing stimulus was applied.

In the present study we analyzed directed networks and neglected weights of directed links. Basically, link weights provide additional information when the total outgoing/incoming information flow of each ROI is evaluated [35], [36]. In a graph theoretical approach, such information is typically estimated through the in- and out-degree of a node [11]. The present study focuses on the estimation of a specific concept which is rather related to the redundancy of the information between ROI pairs. Such concept is mathematically defined by a graph-based index which basically counts the number of multiple paths between nodes, i.e. ROIs. The higher the number of alternative paths is, the higher is the redundancy between two nodes. Indeed, a path is defined as a sequence of links which connects two nodes and it contains topological information describing if and how efficiently two nodes can get in touch. Longer paths imply a higher distance and then a lower efficiency in their communication [37]. It is possible to include link weights into this computation. However, this inclusion is not straightforward as there is not a common consensus on the real meaning of a link weight in the evaluation of path-based distances for functional brain networks [6], [38]. In particular, it is not clear if and to what extend larger weights within a path contribute to shorter or higher "functional" distances between nodes. In this context, some authors have suggested to use the inverse of the weight [11], [39]. Although it appears to be at least an intuitive solution there is no underlying neurobiological evidence for such arbitrary choice. From this point of view, the topological unweighted information between nodes seems to be a more stable approach, and the interpretation of the results is clearer as it can be properly associated to the concept of multiple alternative pathways between brain regions, i.e. redundancy.

We restricted the analysis to the bandwidth with the highest power excluding some interesting bands from analysis (i.e. the gamma band, see e.g. [40]–[42]). The underlying connectivity analysis is based on sensor data. Thus, it is not possible to draw stringent conclusions concerning the involved brain structures due to the relatively low number of electrodes included into the analysis and the difficulty to directly conclude from EEG potentials or connectivity distributions to contributing structures. Therefore we did not primarily interpret our results with respect to neural generators. For such an analysis, a corresponding analysis based on the generator structure would be necessary, see e.g. [43].

In the present study one common order of the underlying AR processes was used. For it the maximum estimated order of the entire data set was selected in order to avoid a model order underestimation. The drawback of this approach is that the common order exceeds the individual order for several sample elements. As described in the context of Granger Causality by Barnett and Seth [44] a model order overestimation may result in an increased amount of unstable AR-parameter estimates, and the effect is even amplified for filtered time series. Definitely a too high model order impairs the AR-parameter estimation quality and has disadvantageous effects to significance tests with respect to Granger Causality indexes [44]. Similar results for PDC analyses may be found in [45].

In our sample, poorer parameter estimates increase the variance of derived network redundancy measures. As a consequence potential group differences might not be revealed. An alternative approach would be to work with individual orders for each sample element. That would improve the AR-parameter estimation quality for several sample elements, and it would not influence the type I error of the significance test transforming raw PDC values into directed interactions. However, it would influence the type II error resulting in different proportions of false negatives in dependence on the model order. From this point of view, the sample elements would not be processed (observed) in the same way, and the influence on a subsequent statistical analysis is unclear. Therefore the first approach was applied being aware of risks and limitations caused by possible overestimated model orders.

Patients were treated with antidepressants. Pain processing in patients treated with norepinephrine and serotonin reuptake inhibitors might be influenced by medication. Thus, future studies need to test the potential influence of this kind of treatment on the results presented here. In conclusion, we found increased network redundancy in HC when compared to patients with MD and our results are suggestive of aberrant pain processing in these patients. Future more detailed investigations focusing on disentangling the network redundancy in regions of the brain involved with processing of noxious stimuli, i.e. in the ”neuromatrix of pain”, are needed.
